# The Presynaptic Scaffold Protein Bassoon in Forebrain Excitatory Neurons Mediates Hippocampal Circuit Maturation: Potential Involvement of TrkB Signalling

**DOI:** 10.3390/ijms22157944

**Published:** 2021-07-26

**Authors:** Anil Annamneedi, Miguel del Angel, Eckart D. Gundelfinger, Oliver Stork, Gürsel Çalışkan

**Affiliations:** 1 Institute of Biology, Otto-Von-Guericke University, 39120 Magdeburg, Germany; delangel@ovgu.de (M.d.A.); oliver.stork@ovgu.de (O.S.); 2 Center for Behavioral Brain Sciences (CBBS), 39120 Magdeburg, Germany; egundelf@lin-magdeburg.de; 3 Leibniz Institute for Neurobiology (LIN), RG Neuroplasticity, 39118 Magdeburg, Germany; 4 Institute of Pharmacology & Toxicology, Medical Faculty, Otto-von-Guericke University, 39120 Magdeburg, Germany

**Keywords:** bassoon, hippocampus, TrkB, neurogenesis, fEPSP, glutamatergic presynapse

## Abstract

A presynaptic active zone organizer protein Bassoon orchestrates numerous important functions at the presynaptic active zone. We previously showed that the absence of Bassoon exclusively in forebrain glutamatergic presynapses (*Bsn^Emx1^*cKO) in mice leads to developmental disturbances in dentate gyrus (DG) affecting synaptic excitability, morphology, neurogenesis and related behaviour during adulthood. Here, we demonstrate that hyperexcitability of the medial perforant path-to-DG (MPP-DG) pathway in *Bsn^Emx1^*cKO mice emerges during adolescence and is sustained during adulthood. We further provide evidence for a potential involvement of tropomyosin-related kinase B (TrkB), the high-affinity receptor for brain-derived neurotrophic factor (BDNF), mediated signalling. We detect elevated TrkB protein levels in the dorsal DG of adult mice (~3–5 months-old) but not in adolescent (~4–5 weeks-old) mice. Electrophysiological analysis reveals increased field-excitatory-postsynaptic-potentials (fEPSPs) in the DG of the adult, but not in adolescent *Bsn^Emx1^*cKO mice. In line with an increased TrkB expression during adulthood in *Bsn^Emx1^*cKO, blockade of TrkB normalizes the increased synaptic excitability in the DG during adulthood, while no such effect was observed in adolescence. Accordingly, neurogenesis, which has previously been found to be increased in adult *Bsn^Emx1^*cKO mice, was unaffected at adolescent age. Our results suggest that Bassoon plays a crucial role in the TrkB-dependent postnatal maturation of the hippocampus.

## 1. Introduction

Transition from adolescence into adulthood requires a substantial remodelling of limbic and cortical circuits (e.g., hippocampus). This fundamental process sets the stage for optimal emotional, cognitive and sexual behaviour during adulthood, deficit of which can lead to neurodevelopmental disorders including autism spectrum disorders or schizophrenia [1,2,3]. Furthermore, environmental stressors during early life can change the course of developmental trajectories of brain circuits and lead to pathological maturation of limbic and cortical circuits [4,5,6]. Thus, proper formation of neuronal circuits (e.g., synaptic pruning) during this period is indispensable for adaptive brain functioning. Of note, synaptic proteins in concert with neurotrophic growth factors such as brain-derived neurotrophic factor (BDNF) and its high-affinity receptor tropomyosin-related kinase B (TrkB) play a fundamental role in brain development and its maturation, which includes the precise and accurate wiring/re-wiring of neuronal circuits.

Bassoon (encoded by the *Bsn* gene), a presynaptic protein involved in the orchestration of the active zone of neurotransmitter release, mediates various functions including proper synaptogenesis and structural development/maturation of the hippocampal *Cornu Ammonis* (CA)3 and dentate gyrus (DG) areas [7,8]. Particularly, Bassoon is fundamental in regulation of neurotransmitter release through interaction with other active zone components like Piccolo, Rab3-interacting molecules (RIMs), RIM-binding proteins (RBPs), Mun13s and ELKS/CAST proteins [9,10,11]. Bassoon is further implicated in regulating presynaptic proteostasis including autophagy and proteasomal pathways [12,13,14].

To date, several genetic mouse models with Bassoon deficiency have been generated. *Bsn* constitutive knockout mice (*Bsn^ΔEx4/5^* and *Bsn^gt^*) suffer from epileptic seizures and sensory processing deficits resulting from loosely anchored ribbons and misplaced voltage-gated Ca2^+^ channels at presynapses of photoreceptors and inner ear hair cells [15,16,17,18]. Distinct electrophysiological alterations are observed in *Bsn^ΔEx4/5^* mice including impaired synaptic fatigue during long-term depression (LTD) induction and long-term potentiation (LTP) at CA1 synapses [19,20]. Recently, we showed that conditional knockout mice for *Bsn* in forebrain excitatory neurons (*Bsn^Emx1^*cKO) display enhanced hippocampal excitability evident by increased baseline transmission at the medial perforant path-to-DG (MPP-DG) and Schaffer collateral (SC)-CA1 synapses without exhibiting a severe epileptic phenotype [8]. These alterations are associated with enhanced learning abilities in hippocampus-dependent tasks including pattern separation and contextual fear conditioning in *Bsn^Emx1^*cKO mice [8]. Thus, Bassoon deficiency appears to have a differential impact on brain function and behaviour in a cell-type specific manner. Together, these observations point to a possible cell type-specific role of Bassoon in circuit maturation (e.g., at hippocampal synapses) affecting cognitive and emotional behaviour later during adulthood.

Of note, the neurotrophic factor BDNF is highly abundant in glutamatergic hippocampal synapses [21] and its signalling through TrkB plays a fundamental role in hippocampal neurogenesis, circuit maturation and synapse elimination in distinct brain regions [22,23,24,25]. Furthermore, altered BDNF/TrkB signalling has been observed in both rodent models [26,27] and patients suffering from autism spectrum disorders and schizophrenia [28,29,30,31]. Intriguingly, hippocampal BDNF levels of *Bsn^ΔEx4/5^* mice are profoundly elevated in adulthood (~12 weeks-old) while only a moderate/insignificant increase in BDNF was detected during adolescence (~4 weeks-old) [32,33]. In addition, TrkB levels appear to be upregulated in the striatum [20] while hippocampal TrkB levels remain unaltered in *Bsn^ΔEx4/5^* mice [32]. These observations indicate that a lack of Bassoon leads to brain region- and age-dependent alterations in the BDNF/TrkB signalling. On the other hand, both *Bsn^Emx1^*cKO and *Bsn^ΔEx4/5^* mice display increased neurogenesis and an enlarged forebrain that becomes particularly significant in the adult hippocampus [8,32,34,35]. This unusual enlargement further points to an abnormal maturation of hippocampal circuits in mice lacking Bassoon potentially triggered by an abnormal BDNF/TrkB signalling.

In the current study, we hypothesized that the increased hippocampal excitability in *Bsn^Emx1^*cKO mice is mediated through enhanced BDNF/TrkB signalling with increasing age. To test this hypothesis, we determined BDNF and TrkB protein levels in the dorsal DG and modulation of baseline synaptic transmission at two hippocampal synapses, i.e., MPP-DG and SC-CA1 synapses, by blocking TrkB in adolescent (~4–5 weeks-old) and adult (~3–5 months-old) *Bsn^Emx1^*cKO mice. While enhanced synaptic excitability could be normalized at adult age, blockade of TrkB signalling did not modulate hippocampal excitability during adolescence. In line with our hypothesis, TrkB levels were aberrantly increased during adulthood but not during adolescence. Our results suggest that *Bsn* deficiency in forebrain excitatory neurons leads to aberrant maturation of hippocampal circuits possibly via an abnormal TrkB-mediated signalling during transition from adolescence to adulthood.

## 2. Results

### 2.1. Increased MPP-DG Synapse Excitability Is Associated with Elevated TrkB Expression and Can Be Normalized by Acute TrkB Blockade in Bsn^Emx1^cKO Mice at Adult Age

**Figure 1:**
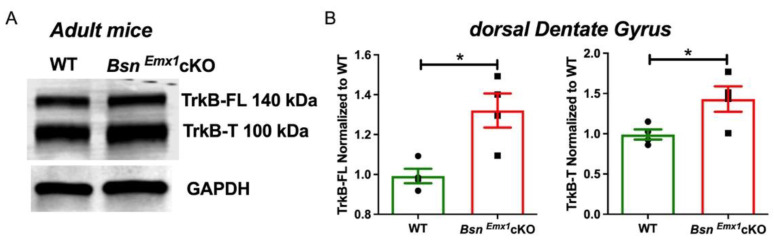
Elevated TrkB levels in the dorsal DG of adult *Bsn^Emx1^*cKO mice (~3–5 months-old). (**A**) Representative western blots of TrkB full length (FL) at ~140 kDa, TrkB truncated (T) form at ~100 kDa and GAPDH, loading control. (**B**) Quantifications of both TrkB isoforms reveals elevated TrkB levels in *Bsn^Emx1^*cKO (*N* = 4 mice) dorsal DG compared to WT mice (*N* = 4 mice). * indicates a significant genotype effect (* *p* < 0.05), Student’s *t* test. All values are expressed as mean ± SEM.

Our previous work demonstrated that a lack of Bassoon in forebrain excitatory neurons results in a general increase in the excitability of both dorsal MPP-DG and SC-CA1 synapses of adult mice [8]. Here, we aimed at identifying possible mechanisms that mediate the increased hippocampal excitability in the *Bsn^Emx1^*cKO mice. We reasoned that increased BDNF/TrkB-mediated signalling as reported before for the constitutive Bassoon mutant (*Bsn^ΔEx4/5^*) mice [32,36] may underlie the increased hippocampal synaptic excitability in the *Bsn^Emx1^*cKO. Thus, we first sought to analyse the levels of BDNF in the dorsal DG using immunohistochemical (IHC) and western blot analyses. However, the levels of BDNF were below detection level in naïve animals in both IHC and immunoblotting. On the other hand, BDNF levels were detected in total Bassoon knockout mice (Bsn KO) as reported previously in *Bsn^ΔEx4/5^* mice [32] (Supplementary Figure S1A,B). These data suggest that upregulation of BDNF is not as prominent as observed in the conventional Bsn constitutive knockout mice. Next, we assessed the TrkB protein levels in the dorsal DG of WT and *Bsn^Emx1^*cKO mice. We identified elevated levels of both full length (FL) and truncated (T) forms of TrkB (TrkB-FL and TrkB-T, respectively) in *Bsn^Emx1^*cKO compared to WT mice (Figure 1A,B; TrkB-FL: T(6) = 3.529, *p* = 0.0124 and TrkB-T: T(6) = 2.583, *p* = 0.0416, Student’s *t* test). We further assessed the synaptic TrkB levels by normalizing both TrkB-FL and TrkB-T to levels of PSD95 (a post synaptic density marker). We did not observe any change in total PSD95 levels between the genotypes (Supplementary Figure S2A; T(6) = 0.1316, *p* = 0.8996). Interestingly, the ratio of TrkB-FL to PSD95 is increased in *Bsn^Emx1^*cKO mice compared to WT mice (Supplementary Figure S2B; T(6) = 2.602, *p* = 0.0406). These data suggest a potential increase in TrkB-mediated signalling via upregulation of synaptic TrkB expression in the DG of *Bsn^Emx1^*cKO mice.

**Figure 2:**
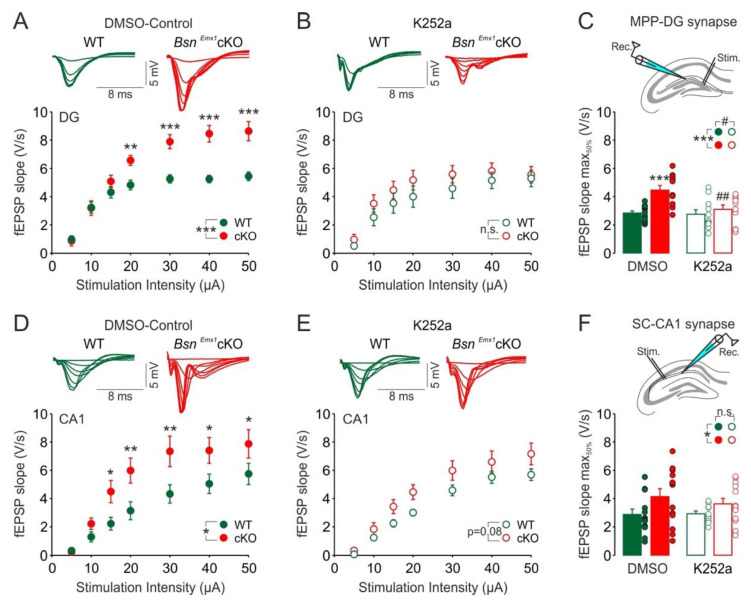
Enhanced hippocampal synaptic excitability in the adult (~3–5 months-old) *Bsn^Emx1^*cKO mice can be normalized by TrkB receptor blockade. Input-Output (I-O) curves showing (**A**) increased field excitatory postsynaptic potential (fEPSP) responses at the medial perforant path-to-dentate gyrus (MPP-DG) synapse of *Bsn^Emx1^*cKO mice and (**B**) its normalization by K252a (200 nM), tyrosine receptor kinase B (TrkB) blocker. (**C**) Two-way ANOVA comparison of fEPSP slopes corresponding to 50% of maximum response revealed a strong genotype and K252a treatment effect at the MPP-DG synapse indicating that TrkB blockade rescues enhanced excitability at the MPP-DG synapse. The positioning of the recording (Rec.) and stimulation (Stim.) electrodes for the MPP-DG synapse is depicted above the summary graph. I-O curves showing (**D**) enhanced fEPSP responses at the Schaffer collateral-to-CA1 (SC-CA1) synapse of *Bsn^Emx1^*cKO mice and (**E**) its partial normalization by TrkB blockade. Note the lack of genotype effect, but a persistent tendency for increased fEPSP responses in the *Bsn^Emx1^*cKO mice in comparison to WT mice under the blockade of TrkB signalling. (**F**) Comparison of fEPSP slopes corresponding to 50% of maximum response shows only a genotype effect without any K252a treatment effect indicating that K252a did not reduce fEPSP slopes significantly at the SC-CA1 synapse of the *Bsn^Emx1^*cKO mice. The positioning of the recording (Rec.) and stimulation (Stim.) electrodes for the SC-CA1 synapse is depicted above the summary graph. Merged traces to increasing stimulation strengths (5–50 µA) for each condition are depicted above the I-O curves (WT: Green; *Bsn^Emx1^*cKO: Red). Slices were pre-treated (2 h) either with vehicle DMSO (DG-WT: *N* = 4 mice, *n* = 13 slices; DG-*Bsn^Emx1^*cKO: *N* = 4 mice, *n* = 11 slices; CA1-WT: *N* = 4 mice, *n* = 13 slices; CA1-*Bsn^Emx1^*cKO: *N* = 4 mice, *n* = 13 slices) or K252a (DG-WT: *N* = 4 mice, *n* = 11 slices; DG-*Bsn^Emx1^*cKO: *N* = 4 mice, *n* = 11 slices; CA1-WT: *N* = 4 mice, *n* = 13 slices; CA1-*Bsn^Emx1^*cKO: *N* = 4 mice, *n* = 10 slices). * indicates a significant genotype effect (* *p* < 0.05; ** *p* < 0.01: *** *p* < 0.001) and # indicates a significant K252a treatment effect (# *p* < 0.05; ## *p* < 0.01). n.s. indicates not significant. Two-way repeated ANOVA followed by posthoc comparison using Fisher LSD Method was performed for (**A**,**B**,**D**,**E**). Two-way ANOVA followed by posthoc comparison using Fisher LSD Method was performed for (**C**,**F**). All values are expressed as mean ± SEM.

To test whether the enhanced TrkB expression in the dorsal DG of *Bsn^Emx1^*cKO mice is associated with an altered TrkB-mediated modulation of synaptic excitability in the dorsal DG, we recorded input-output (I-O) curves via measuring fEPSP responses at the MPP-DG synapse to increasing stimulation strengths with or without pre-application of TrkB blocker, K252a (Figure 2). We first confirmed the previously reported [8] increased excitability at the MPP-DG synapse (Figure 2A, F(1, 22) = 15.863, *p* < 0.001) under control-DMSO condition. There was a significant genotype × stimulation intensity interaction (F(1, 6) = 10.440, *p* < 0.001) revealing genotype differences for stimulation intensities above 15 µA (Fisher LSD Method, 20 µA: *p* = 0.004, 30–50 µA: *p* < 0.001). Interestingly, no genotype differences were observed for slices treated with K252a (Figure 2B, F(1, 20) = 1.022, *p* = 0.324) indicating a possible rescue of enhanced synaptic excitability in the DG of *Bsn^Emx1^*cKO. Two-way ANOVA comparison of half maximal fEPSP slope values for each I-O curve (Figure 2C) revealed both genotype (F(1, 42) = 12.693, *p* < 0.001) and treatment (F(1, 42) = 6.835, *p* = 0.012) differences with a significant genotype x treatment interaction (F(1, 42) = 5.325, *p* = 0.026). Posthoc comparison revealed a genotype effect only under control-DMSO condition (*p* < 0.001) and a K252a-mediated reduction in fEPSP slopes only in *Bsn^Emx1^*cKO mice (*p* = 0.001). These data demonstrate the acute rescue of elevated MPP-DG synapse excitability via blockade of TrkB-mediated signalling in the *Bsn^Emx1^*cKO mice.

Similarly, we identified an increase in the excitability of SC-CA1 synapse under control-DMSO condition (Figure 2D, F(1, 24) = 5.390, *p* = 0.029) with a significant genotype x stimulation intensity interaction (F(1, 6) = 3.654, *p* = 0.002) revealing genotype differences for stimulation intensities above 10 µA (Fisher LSD Method, 15 µM: 0.026, 20 µA: *p* = 0.006, 30 µA: *p* = 0.004, 40 µA: *p* = 0.021, 50 µA: *p* = 0.037). Under k252a pre-treatment, the genotype difference remained only as a statistical trend (Figure 2E, F(1, 24) = 3.487, *p* = 0.076). Last, comparison of half maximal fEPSP slopes revealed only a general genotype difference (Figure 2F, F(1, 45) = 5.339, *p* = 0.025) without any treatment effect (Figure 2F, F(1, 45) = 0.301, *p* = 0.586) and treatment x genotype interaction (Figure 2F, F(1, 45) = 0.438, *p* = 0.511). In sum, these data indicate that synaptic hyperexcitability observed at the DG-MPP is a result of elevated TrkB levels and can be rescued by the blockade of TrkB-mediated signalling whereas the impact of this intervention is minimal in reducing hyperexcitability at the SC-CA1 synapse of *Bsn^Emx1^*cKO mice.

### 2.2. Enhanced Hippocampal Excitability and Its Modulation by TrkB-Mediated Signalling in the Bsn^Emx1^cKO Mice Are Not Evident during Adolescence

**Figure 3:**
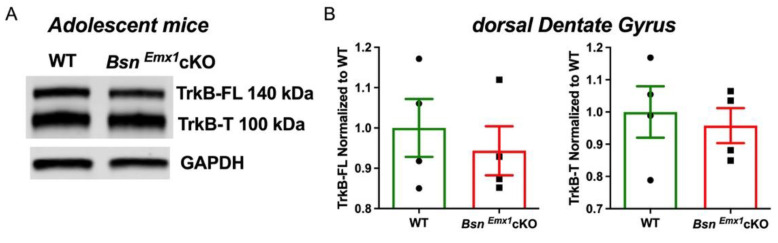
Unaltered TrkB levels in the dorsal DG of adolescent *Bsn^Emx1^*cKO mice (~4–5 weeks-old). (**A**) Representative western blots of TrkB full length (FL), TrkB truncated (T) form and a loading control, GAPDH. (**B**) Quantifications of both the TrkB isoforms reveal no change in TrkB levels of adolescent *Bsn^Emx1^*cKO (*N* = 4 mice) dorsal DG compared to WT mice (*N* = 4 mice). Student’s *t* test. All values are expressed as mean ± SEM.

Previous observations from the *Bsn^ΔEx4/5^* mice suggest that hippocampal BDNF levels are moderately elevated at adolescent age (~4 weeks old) but reach significantly high levels only at adult stage [32]. Thus, to test whether such postnatal regulation of hippocampal synaptic excitability via TrkB-mediated signalling is also present in the *Bsn^Emx1^*cKO mice, we performed analogous immunoblot and electrophysiological experiments using adolescent mice (~4–5 weeks old). We assessed both TrkB-FL and TrkB-T levels in the dorsal DG using quantitative western blot analysis in adolescent WT and *Bsn^Emx1^*cKO mice. No changes were observed between the genotypes (Figure 3A,B; TrkB-FL: T(6) = 0.600, *p* = 0.5703 and TrkB-T: T(6) = 0.439, *p* = 0.6757, Student’s *t* test) suggesting a potential upregulation of TrkB signalling only during the transition from adolescence to adulthood.

**Figure 4:**
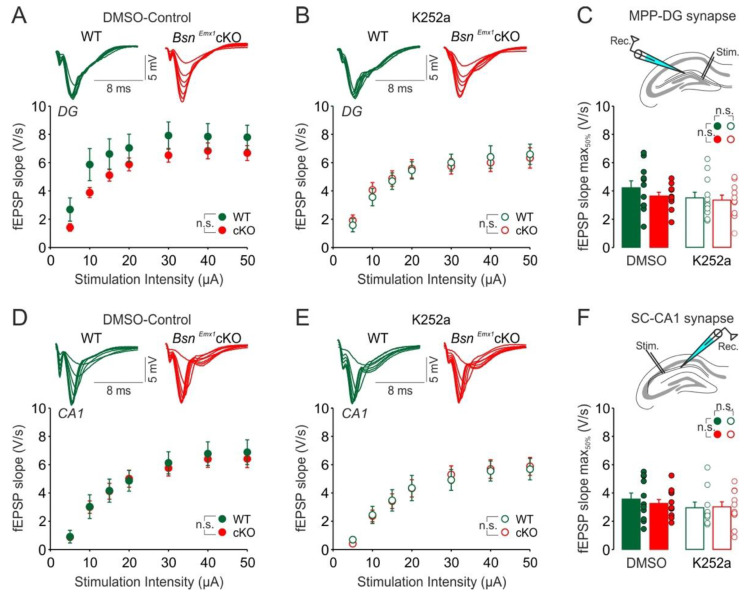
Hippocampal synaptic excitability in the *Bsn^Emx1^*cKO mice is not altered during adolescence (~4–5 weeks-old). Input-Output (I-O) curves showing (**A**) no significant alterations in the fEPSP responses at the MPP-DG synapse of *Bsn^Emx1^*cKO mice under control conditions and (**B**) under the blockade of TrkB receptors by K252a (200 nM). (**C**) Two-way ANOVA comparison of fEPSP slopes corresponding to 50% of maximum response revealed no genotype and no K252a treatment effect at the MPP-DG synapse. The positioning of the recording (Rec.) and stimulation (Stim.) electrodes for the MPP-DG synapse is depicted above the summary graph. I-O curves showing (**D**) no alterations in the fEPSP responses at the SC-CA1 synapse of *Bsn^Emx1^*cKO mice under control conditions and (**E**) after k252a. (**F**) Comparison of fEPSP slopes corresponding to 50% of maximum response shows no genotype and no treatment effect at the SC-CA1 synapse. The positioning of the recording (Rec.) and stimulation (Stim.) electrodes for the SC-CA1 synapse is depicted above the summary graph. Merged traces to increasing stimulation strengths (5–50 µA) for each condition are depicted above the I-O curves (WT: Green; *Bsn^Emx1^*cKO: Red). Slices were pre-treated (2 h) either with vehicle DMSO (DG-WT: *N* = 5 mice, *n* = 11 slices; DG-*Bsn^Emx1^*cKO: *N* = 5 mice, *n* = 11 slices; CA1-WT: *N* = 5 mice, *n* = 11 slices; CA1-*Bsn^Emx1^*cKO: *N* = 5 mice, *n* = 12 slices) or K252a (DG-WT: *N* = 5 mice, *n* = 12 slices; DG-*Bsn^Emx1^*cKO: *N* = 5 mice, *n* = 11 slices; CA1-WT: *N* = 5 mice, *n* = 10 slices; CA1-*Bsn^Emx1^*cKO: *N* = 5 mice, *n* = 12 slices). n.s. indicates not significant. Two-way repeated ANOVA followed by posthoc comparison using Fisher LSD Method was performed for (**A**,**B**,**D**,**E**). Two-way ANOVA followed by posthoc comparison using Fisher LSD Method was performed for (**C**,**F**). All values are expressed as mean ± SEM.

We further observed no change in the I-O curves under both DMSO-control (Figure 4A, F(1, 20) = 1.876, *p* = 0.186) and K252a (Figure 4B, F(1, 21) = 0.002, *p* = 0.968) conditions at the MPP-DG synapse of *Bsn^Emx1^*cKO mice. Comparison of half maximal fEPSP slope values (Figure 4C) revealed also no genotype (F(1, 41) = 0.914, *p* = 0.345) or treatment (F(1, 41) = 1.666, *p* = 0.204) effect. Similarly, for the hippocampal CA1 subregion, no change in the I-O curves under both DMSO-control (Figure 4D, F(1, 21) = 0.042, *p* = 0.804) and K252a (Figure 4E, F(1, 20) = 0.002, *p* = 0.961) conditions was detected. Half maximal fEPSP slope values (Figure 4F) were also not affected (genotype: F(1, 41) = 0.107, *p* = 0.745; treatment: F(1, 41) = 1.321, *p* = 0.257). Together, these data suggest that hippocampal hyperexcitability and its interaction with TrkB-mediated signalling is only evident in adult *Bsn^Emx1^*cKO mice as no changes were detected at this young age.

### 2.3. Unaltered Neurogenesis in Adolescent Bsn^Emx1^cKO Mice

**Figure 5:**
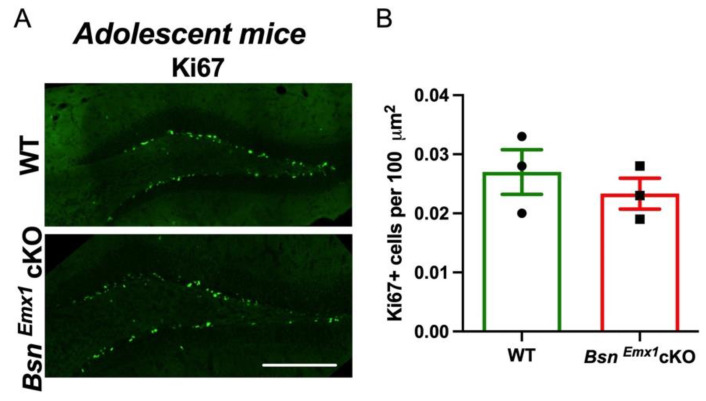
Neurogenesis is not altered in adolescent (~4–5 weeks-old) *Bsn^Emx1^*cKO mice. (**A**) Representative hippocampus sections from WT (*N* = 3 mice, *n* = 3–4 sections per animal) and *Bsn^Emx1^*cKO (*N* = 3 mice, *n* = 4 sections per animal) showing cells positive for proliferation marker Ki67 (Ki67+) in the DG. Scale bar is 250 µm. (**B**) Quantification of Ki67+ cells in the suprapyramidal blade of the DG granule cell layer display no differences between WT and *Bsn^Emx1^*cKO mice. Student’s *t* test. All values are expressed as mean ± SEM.

We previously showed that *Bsn^Emx1^*cKO mice displayed increased neurogenesis during adulthood [8]. To further evaluate the effects of Bassoon loss on the rate of neurogenesis during adolescence, we performed immunohistochemical analysis of Ki67, a proliferative marker using 4–5 weeks-old mice (Figure 5A). We found no changes in the number of cells that are positive for Ki67 between WT and *Bsn^Emx1^*cKO (Figure 5B, t(4) = 0.7980, *p* = 0.4696, Student’s *t* test), indicating that Bassoon deficiency in forebrain excitatory neurons does not influence neurogenesis at adolescent age.

## 3. Discussion

In the current study, we aimed at gaining insights into the potential signalling mechanisms behind the synaptic hyperactivity in the hippocampus of adult *Bsn^Emx1^*cKO mice. We postulated that aberrant changes in the BDNF/TrkB signalling might underlie the observed hippocampal phenotype. Our electrophysiological analysis confirms a generally enhanced excitability at two hippocampal synapses (MPP-DG and SC-CA1) in adult, but not in young adolescent *Bsn^Emx1^*cKO mice. Intriguingly, TrkB protein levels (both full length and truncated forms) are elevated in the dorsal DG of adult *Bsn^Emx1^*cKO mice (Figure 1). In line with an upregulation of synaptic TrkB levels (Figure 1; Supplementary Figure S2), acute blockade of TrkB signalling normalizes the synaptic hyperexcitation at the MPP-DG synapse in the *Bsn^Emx1^*cKO mice. This happens only at adult age without any effect at adolescent age, consistent with unaltered TrkB levels in adolescence (Figure 2, Figure 3 and Figure 4). Accordingly, neurogenesis during adolescence was not affected in *Bsn^Emx1^*cKO mice (Figure 5). Together, our study indicates that Bassoon deficiency in forebrain excitatory neurons leads to aberrant postnatal maturation of the hippocampus that can be normalized via a TrkB signalling blockade.

In the previous study, we focused on the impact of *Bsn* deficiency in forebrain excitatory neurons on neuronal function, neurogenesis, hippocampal physiology and behaviour during adulthood. We showed that adult *Bsn^Emx1^*cKO mice display enhanced baseline transmission at the MPP-DG synapses, increased granule cell dendritic complexity, enlarged brain structures, enhanced neurogenesis, increased numbers of immature granule cells and DG-dependent learning [8]. We noted that the enhanced synaptic excitability in the DG might underlie such a gain-of-function. While these findings provide important insight into the role of Bassoon in regulation of hippocampal function and hippocampus-dependent learning and memory, pathogenesis of such altered functions remained elusive.

In this framework, we noted a delayed maturation of the DG circuit evident by sustained high synaptic excitability across development together with an increased number of immature neurons at adult stage [8]. Several lines of evidence indicate that elevated BDNF/TrkB signalling enhances synaptic function and neurogenesis in the hippocampus and overexpression of TrkB-T elevates proliferation, per se [37,38,39,40]. Thus, a sustained enhancement of BDNF/TrkB signalling via upregulation of either the neurotrophic growth factor BDNF or its high affinity receptor TrkB can potentially lead to a lasting increase in the synaptic function. Accordingly, TrkB KO mice show reduced hippocampal maturation and synaptogenesis [25] and conditional ablation of TrkB in forebrain excitatory neurons which leads to a deficit in hippocampal synaptic plasticity and hippocampus-dependent behaviour [41]. In line with these findings, pharmacological treatment with the TrkB blocker (K252a) could normalize behavioural and physiological alterations in the *Bsn^ΔEx4/5^* mice [36]. Thus, we assessed the impact of subacute blockade of TrkB signalling on synaptic transmission in the MPP-DG and SC-CA1 pathways using acute hippocampal slice preparations of *Bsn^Emx1^*cKO mice. Indeed, the enhanced synaptic excitability in the DG could be normalized by TrkB blockade, suggesting that elevated BDNF/TrkB signalling might cause the enhanced synaptic excitability in the DG of *Bsn^Emx1^*cKO. On the other hand, only a partial rescue was evident in the SC-CA1 synapse suggesting that additional factors such as neurogenic circuit properties, which are enhanced in the DG of adult *Bsn^Emx1^*cKO mice, might tune the impact of TrkB blockade on synaptic function. K252a pre-treatment did not alter synaptic excitability of MPP-DG in both WT and *Bsn^Emx1^*cKO adolescent mice. Accordingly, no change in the TrkB levels (neither full length nor truncated forms) and rate of neurogenesis was detected between the genotypes at adolescent age (4–5 weeks-old). These results suggest that presynaptic Bassoon plays a critical role in postnatal maturation of hippocampal circuits via an interplay with TrkB signalling.

Previously reported BDNF/TrkB-associated behavioural and morphological phenotypes remarkably resemble the phenotype of *Bsn^Emx1^*cKO mice [8]. In line with the enhanced performance in pattern separation in *Bsn^Emx1^*cKO mice, BDNF activity in the DG is essential for discrimination of very similar spatial representations [42]. Furthermore, BDNF action on adult born immature granule cells via TrkB receptors appears to be necessary for discrimination of similar memories [43]. In addition, BDNF/TrkB signalling plays an important role in contextual fear conditioning, evident by a deficit in contextual fear memory in heterozygous BDNF^+/−^ mice and its partial recovery by BDNF infusion [44]. Similarly, over expression of full length TrkB leads to enhanced fear memory in mice [45]. Last, but not least, overexpression of BDNF in the DG leads to increased dendritic complexity through TrkB receptors as observed in the *Bsn^Emx1^*cKO mice [46] while a lack of TrkB receptors reduces the dendritic complexity in cortical areas [47]. Such aberrant alterations in circuit maturation and BDNF/TrkB signalling are profoundly evident in neurodevelopmental disorders including autism spectrum disorders (ASDs) and schizophrenia [1,2,3,28,29,30,31]. Thus, our findings conform with these observations and call for future studies investigating potential interaction of Bassoon and BDNF/TrkB-mediated signalling in the pathogenesis of brain disorders that are associated with aberrant circuit maturation (e.g., synaptic pruning).

What could be the possible mechanism behind the increased TrkB signalling in adult *Bsn^Emx1^*cKO mice? A potential mechanism might be that the impaired maturation of the DG, as evidenced by increased neurogenesis and immature granule cells, i.e., doublecortin positive (DCX+) cells in the *Bsn^Emx1^*cKO [8], keeps TrkB levels up during circuit maturation. Two studies have reported that TrkB is expressed in proliferating and immature granule cells like Ki67 and DCX+, respectively [48,49]. Thus, the elevated TrkB levels in *Bsn^Emx1^*cKO mice might be a consequence of a lack of DG maturation evident by sustained synaptic hyperexcitability and neurogenesis across development in the absence of Bassoon. Of note, we did not observe any effects of K252a treatment on synaptic excitability in adolescent *Bsn^Emx1^*cKO mice. In addition, the levels of TrkB in dorsal DG and expression of proliferative marker Ki67 were unaffected at this age. These findings suggest that Bassoon in excitatory neurons might be particularly involved in post adolescent adult born neurogenesis. Furthermore, our assessment of synaptic marker PSD95 suggests that the increased TrkB levels might not be due to an overall increase in the synapse density but rather an increase in the synaptic TrkB levels per se in the DG of *Bsn^Emx1^*cKO mice.

Intriguingly, *Bsn^Emx1^*cKO mice show a differential phenotype in comparison to the conventional mutants with a complete lack of Bassoon function in the brain (e.g., *Bsn^ΔEx4/5^*). This might be due to severe epileptic seizures in *Bsn^ΔEx4/5^* mice that might further exacerbate BDNF levels [50]. This is highly unlikely in *Bsn^Emx1^*cKO mice which are essentially free of severe epileptic seizures [8]. In line, our immunohistochemical and western blot analysis could not detect a measurable BDNF immunoreactivity in both WT and *Bsn^Emx1^*cKO mice, indicating that a pathological upregulation of BDNF levels as observed in the constitutively Bassoon-mutant mice does not occur to a similar extent in the *Bsn^Emx1^*cKO mice. Interestingly, lack of Bassoon only in excitatory neurons appears to have rather improving effects on hippocampus-dependent memory possibly due to enhanced hippocampal synaptic transmission [8]. Due to severe epileptic seizures, studying such functions is not feasible in *Bsn^ΔEx4/5^* mice. Thus, the *Bsn^Emx1^*cKO mouse provides an excellent model to study the functions of Bassoon in the absence of severe repetitive epileptic seizures and potential secondary processes.

Our findings together with previous reports further call for studies that elucidate the impact of Bassoon deficiency in brain function in a cell-type specific manner. Particularly, there is a clear lack of knowledge on the impact of Bassoon deficiency in different types of interneurons despite the importance of BDNF/TrkB signalling for their function and their crucial role in circuit maturation [51,52]. Nevertheless, our study suggests a critical role for Bassoon in excitatory forebrain neurons and its interaction with TrkB signalling in hippocampal circuit maturation.

## 4. Materials and Methods

### 4.1. Animals

**Figure 6:**
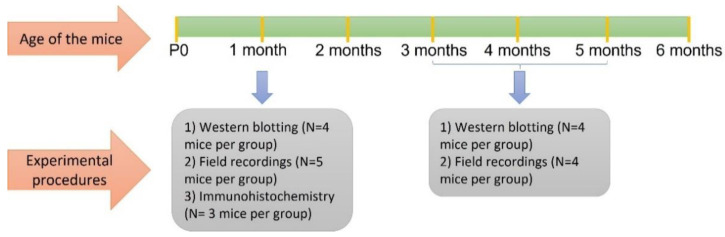
The timeline of experimental procedures together with the number of animals used for each experimental protocol.

Conditional knockout mice lacking Bassoon in excitatory neurons (*Bsn^Emx1^*cKO) were used in this study. *Bsn^Emx1^*cKO mice were generated by crossing mice, in which exon 2 of the Bsn gene was floxed, with mice expressing Cre recombinase under the control of the Emx1 promoter (Bsn2^lx/lx^ /Emx1^Cre/+^). Generation and genotyping of the *Bsn^Emx1^*cKO mice was performed as described previously [8]. The constitutive Bassoon knockout mice (Bsn KO) used in this study were generated as described previously [53]. Adult male (unless mentioned otherwise) *Bsn^Emx1^*cKO mice and littermates or age matched wild-type mice (WT) aged between ~3–5 months and adolescent mice (~4–5 weeks-old) were used. In total, 40 mice were used in the study (Adult WT: 8 mice; Young WT: 12 mice; Adult *Bsn^Emx1^*cKO: 8 mice; Young *Bsn^Emx1^*cKO: 12 mice). Figure 6 provides the order and the timeline of experimental procedures. The experimenter was blind to the genotype. The genotype of the mice was only revealed after the complete data analysis was performed. Breeding was performed at the Leibniz Institute for Neurobiology, Magdeburg and test animals were obtained using breeding scheme Bsn2^lx/lx^Emx1^Cre/+^ × Bsn2^lx/lx^Emx1^+/+^. Mice from the same sex were group housed in a type II cages with food, water ad libitum. All the experiments performed in this study were conducted during dark phase from 9:30 a.m. to 5 p.m. (12 hr dark/light cycle; lights off at 7:30 a.m.) and in accordance with the European and German regulations for animal experiments and were approved by Landesverwaltungsamt Sachsen-Anhalt (License number: 42502-2-1303 LIN/-1484 LIN).

### 4.2. Immunohistochemistry and Microscopy

Immunohistochemical (IHC) procedure including perfusion of the adoloscent mice was perfomed as described in detail previously [8]. Briefly, transcardial perfusion on anesthetized mice was perfomed using 4%PFA. Brains were then dissected carefully and post fixed in 4% PFA, cryoprotected in 30% sucrose in phosphate buffer saline (PBS) solution and stored at −80 °C until utilized. 30 μm thick coronal sections were cut on a cryostat and free floating sections were used for immunological stainings. Free-floating sections were incubated with blocking solution (10% normal goat serum (NGS) and 0.3% Triton X-100 in PBS) for 1 h at room temperature. Sections were then incubated with primary antibody in the same blocking solution overnight at 4 °C. Sections were then washed in PBS (three times, 10 min each) followed by overnight incubation with appropriate secondary antibodies at 4 °C. Finally, post washing, sections were mounted on glass slides and covered with coverslips using fluoromount g DAPI (Southern biotech, USA), which is used for nuclear counterstaining. Antibodies used for IHC include primary antibodies-rabbit anti-Ki67 (#ab15580, Abcam, Cambridge, United Kingdom, UK, RRID:AB_443209) (1:500), mouse anti-BDNF (3C11) (#ab203573, Abcam, Cambridge, UK, AB_2631315) (1:350), rabbit anti-Piccolo (homemade) (1:500) and secondary antibodies coupled with fluorophores include donkey anti-rabbit Alexa 488 (#A21206, Invitrogen, California, USA, RRID:AB_141708) (1:500), anti-mouse Alexa 488 (#A21202, Invitrogen, RRID:AB_141607) (1:500) and anti-rabbit Cy3 (#711-165-152, Jackson Immuno Research Labs, Pennsylvania, USA, RRID:AB_2307443) (1:500).

Ki67 staining analysis and the imaging of dorsal DG images with BDNF/Piccolo IHC was performed as described previously [8], with minor modifications. A Leica microscope (40× Dry objective, NA 0.6, LAS X software with THUNDER technology) was used to acquire the Z-stack images (20 µm stack volume) of the whole area spanning the dentate gyrus. Image-J was used to track the granule cell layer using DAPI labeling and Ki67 positive cells were counted using a cell counter tool within the granule cell layer and normalized to the area.

### 4.3. Tissue Preparation for Western Blot Analyses

Adult mice between 4–5 months of age and adolescent mice between 4–5 weeks of age (mixed sex) were used for western blot analyses. Tissue was dissected as described previously [54]. Briefly, each hemisphere was separated by the longitudinal fissure using a sterile surgical blade, then the brainstem and the cerebellum were removed to expose the hippocampus. Using a 30 mm gauge sterile needle, the dentate gyrus (DG) was dissected from the hippocampus by making a longitudinal cut between the DG and the CA3 area. Further, DG was divided into dorsal and ventral areas by making a cut in the middle with a surgical blade and dorsal DG was collected for further analysis. Dorsal DG samples from one hemisphere were snap frozen on liquid nitrogen and then stored at −80 °C for quantitative western blotting.

For quantitative western blot analysis the dorsal DG samples were homogenized using a sterile pestle in cold LM (n-Dodecyl-β-D-Maltoside) buffer (50 µL each) and then incubated at 4 °C for 25 min. Samples were centrifuged at 13,000 G for 10 min and the supernatant was collected for protein analysis using Bio-Rad DC protein assay. 20 μg protein per lane were loaded onto Bio-Rad Any kD™ Mini-PROTEAN^®^ Precast Protein Gels and transferred onto Immobilon-FL PVDF membranes (Millipore). Blots were incubated with primary antibodies-mouse anti-BDNF (3C11) (1:1000), goat anti-TrkB (#AF1494, R&D Systems, Inc., Minneapolis, USA, RRID: AB_2155264) (1:1000), mouse anti-PSD95 (#75-028, NeuroMab, California, USA, RRID:AB_2292909) (1:2500), rabbit anti-GAPDH (D16H11) (#5174, Cell Signaling Technology, Massachusetts, USA, AB_10622025) (1:1000) in TBS containing 5% BSA, 0.1% Tween and 0.025% sodium azide at 4 °C overnight and with secondary antibodies including goat anti-rabbit IgG IRDye 680 (#926-68071, LI-COR, Nebraska USA, RRID:AB_10956166), anti-goat IgG IRDye 800 (#926-32214, LI-COR, RRID:AB_621846), anti-mouse IgG IRDye 800 (#827-08364, LI-COR, RRID:AB_10793856) and anti-mouse IgG IRDye 680 (#926-32222, LI-COR, RRID:AB_621844) (1:10000) in TBS containing 3% BSA, 0.1% Tween either at 4 °C overnight or at 1.5–2 h at room temperature. Integrated density signals were quantified by marking identical ROIs around the bands using an Odyssey Infrared Scanner (LI-COR) and Image Studio software. ID values were normalized to loading control GAPDH and further to mean value of the WT group and expressed as normalized values.

### 4.4. Slice Preparation for Electrophysiology

Electrophysiology experiments were performed as reported in our previous study [8]. Mice were decapitated under deep isoflurane anesthesia. The brain was extracted and immersed in a carbogenated (5% CO_2_/ 95% O_2_) artificial cerebrospinal fluid (aCSF; 4–8 °C, pH 7.4, osmolarity ~300 mosmol/kg) with the following ingredients: (in mM) 129 NaCl, 21 NaHCO_3_, 3 KCl, 1.6 CaCl_2_, 1.8 MgSO_4_, 1.25 NaH_2_PO_4_ and 10 glucose. To obtain dorsal transverse-like hippocampal sections, 400 µM parasagittal brain slices were cut from the septal pole using a platform with a 12° angle [55]. Slices were placed in an interface chamber perfused with aCSF at 34.0 ± 1.0 °C (flow rate: 2.0 ± 0.2 mL/min). At least 1 h was allowed for slice recovery before slices were divided into two groups to be either perfused with the vehicle dimethyl sulfoxide (DMSO (0.1%); Tocris, Bristol, UK) or TrkB receptor blocker K252a (200 nM; Tocris, Bristol, UK). The genotype of mice was revealed only after the end of each experimental batch.

### 4.5. Extracellular Field Potential Recordings

Two hours after perfusion with the vehicle DMSO or K252a, slices were placed into the recording interface chamber for extracellular field recordings. To obtain recordings from the medial perforant path (MPP) to dentate gyrus (DG) synapse, the recording glass electrode filled with aCSF (~1 MΩ) was placed at the mid-molecular layer at 70–100 µm depth. On the other hand, a bipolar tungsten wire electrode (exposed tips: ~20 µm; tip separations: ~75 µm; electrode resistance in aCSF: ~0.1 MΩ) was used for stimulation of MPP by placing it to the middle one third of the molecular layer. The main characteristics of the MPP-DG synapse, a paired-pulse depression at 50 ms inter-pulse interval, was confirmed for each slice recording [56,57]. On the other hand, to obtain recordings from the Schaffer collateral (SC) to Cornu Ammonis (CA)1 synapse, the recording electrode was placed at the stratum radiatum (SR) of the CA1 subregion whereas the stimulation electrode was placed at the proximal CA1. For each synapse, baseline responses were recorded for a duration of 5–10 min (0.033 Hz, pulse duration: 100 µs) to ensure the stability of the responses. Then, an input-output (I-O) curves with varying stimulation strengths (in µA: 5, 10, 15, 20, 30, 40, 50) were obtained. A custom-made amplifier was used to pre-amplify and low-pass filter (<3 kHz) the extracellular signal recorded. The data was sampled at 10 kHz (Spike 2.8., Cambridge Electronic Design Limited, UK) and stored on a computer for off-line analysis.

### 4.6. Data Analysis and Statistics

Self-written MATLAB-based analysis tools were used for off-line analysis of field excitatory postsynaptic potential (fEPSP) responses (MathWorks, Natick, MA). The slope between 20 and 80% of the maximum fEPSP amplitude was measured for each stimulation strength and I-O curves were obtained for each slice recorded (2–4 slices per condition (DMSO vs. K252a) per mouse). To determine genotype (WT vs. *Bsn^Emx^*cKO) differences under control-DMSO or K252a condition, I/O curves were compared using two-way repeated measures ANOVA followed by posthoc comparison using the Fisher LSD Method. To determine genotype and treatment (DMSO vs. K252a) effects, maximum slope value from each I-O curve was extracted. Half maximum slope values were statistically compared using two-way ANOVA followed by posthoc comparison using the Fisher LSD Method (SigmaPlot for Windows Version 11.0, 2008 (Systat software GmbH, Erkrath). It should be noted that part of the control data presented in the current study was published in our previous work [8]. Immunohistochemical and western blot data were analysed and graphs were plotted using MS-Office Excel (MS Office 365) and GraphPad Prism (version 9, USA). For group comparison, data was evaluated either by the unpaired Student’s *t* test or the Mann-Whitney U test based on the outcome of the Shapiro-Wilk normality test. No statistical tests were performed to predetermine the sample sizes of the number of mice. However, the sample sizes are similar to those reported previously [8]. No outliers were detected in the statistical analysis. Accordingly, none of the experimental animals were excluded from the study.

## Data Availability

The data presented in this study are available on request from the corresponding authors.

## Supplementary Materials

The following are available online at https://www.mdpi.com/article/10.3390/ijms22157944/s1.
