# Supplementary material for: The Presynaptic Scaffold Protein Bassoon in Forebrain Excitatory Neurons Mediates Hippocampal Circuit Maturation: Potential Involvement of TrkB Signalling
Source: Int J Mol Sci. 2021 Jul 26;22(15):7944. doi: 10.3390/ijms22157944 (PMC8347324; doi:10.3390/ijms22157944)
Supplement: Supplementary file 1 [file ijms-22-07944-s001.zip › ijms-1310950-supplementary.pdf]

Supplementary Material

The Presynaptic Scaffold Protein Bassoon in Forebrain Excitatory Neurons Mediates Hippocampal Circuit Maturation: Potential Involvement of TrkB Signalling

Anil Annamneedi [1,2,3,*], Miguel del Angel [1], Eckart D. Gundelfinger [2,3,4], Oliver Stork [1,2] and Gürsel Çalışkan [1,2,*]

[1] Institute of Biology, Otto-von-Guericke University, 39120 Magdeburg, Germany
[2] Center for Behavioral Brain Sciences (CBBS), 39120 Magdeburg, Germany
[3] Leibniz Institute for Neurobiology (LIN), 39118 Magdeburg, Germany
[4] Institute of Pharmacology & Toxicology, Medical Faculty, Otto-von-Guericke University, 39120 Magdeburg, Germany
* Correspondence: guersel.caliskan@ovgu.de (G.C.); anil.annamneedi@ovgu.de (A.A.)

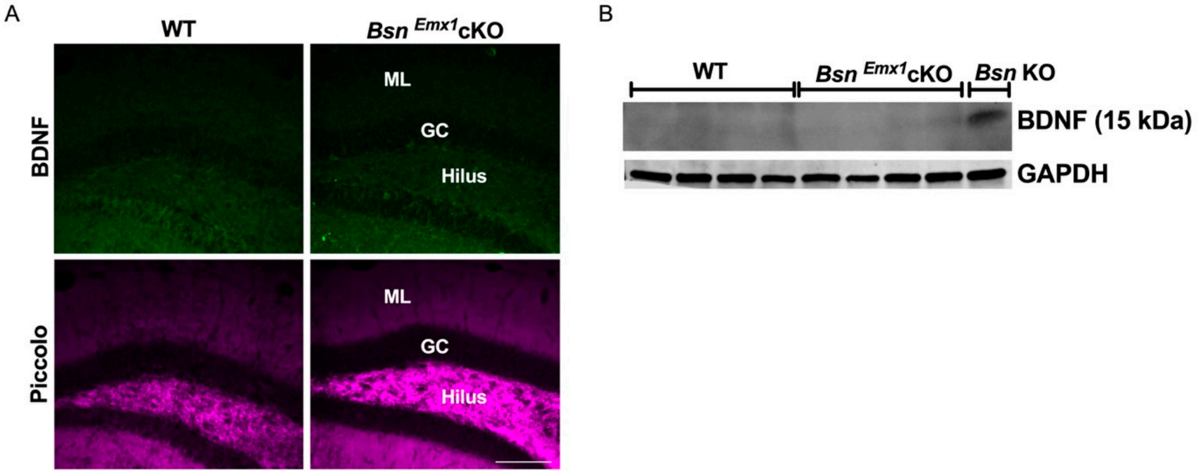

Supplementary Figure S1. Undetectable BDNF levels in the dorsal DG of adult Bsn ^{Emx1}cKO mice**.** (**A**) Dorsal DG sections were stained with BDNF antibody reveal no detectable BDNF immunoreactivity above background in granule cell (GC) layer and molecular layer (ML) of DG. Lower panel showing Piccolo immunoreactivity as a reference. Scale bar is 150 µm. (**B**) Representative western blot of BDNF showing detectable band at 15 kDa only in Bsn KO and GAPDH, loading control.

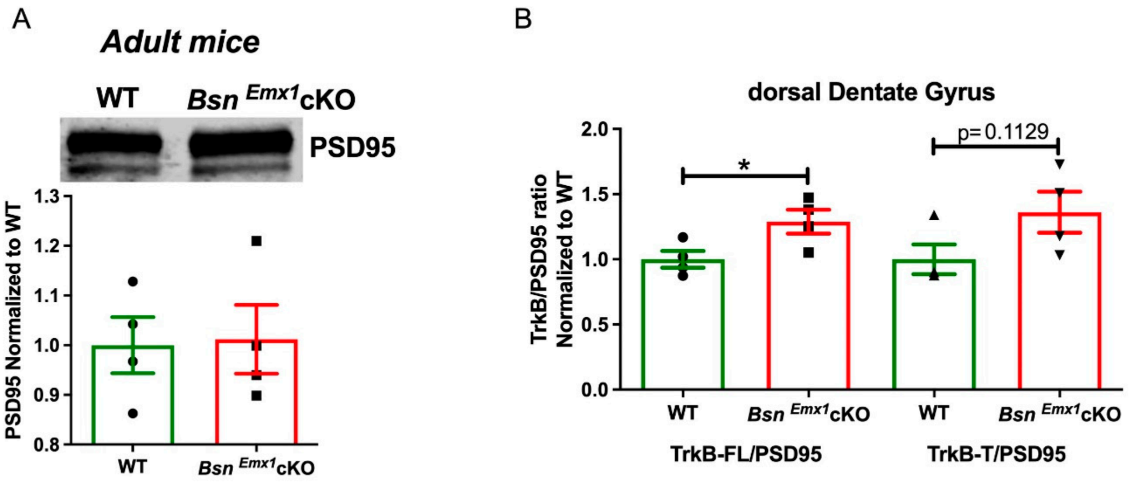

Supplementary Figure S2. Elevated synaptic TrkB levels in the dorsal DG of adult *Bsn* ^{Emx1}cKO mice. (**A**) Representative western blot of PSD95 at ~95–110 kDa. Quantification of total PSD95 reveals no change in *Bsn* ^{Emx1}cKO (N = 4 mice) dorsal DG compared to WT mice (N = 4 mice). (**B**) Levels of both TrkB FL and T isoforms reveals a significance TrkB-FL vs PSD95 ratio in *Bsn*^{Emx1}cKO compared to WT mice, suggesting an elevated synaptic TrkB expression. * indicates a significant genotype effect (* $p <$ 0.05), Student's t test. All values are expressed as mean ± SEM.
